# Nanoparticle Systems for Cancer Phototherapy: An Overview

**DOI:** 10.3390/nano11113132

**Published:** 2021-11-20

**Authors:** Thais P. Pivetta, Caroline E. A. Botteon, Paulo A. Ribeiro, Priscyla D. Marcato, Maria Raposo

**Affiliations:** 1 CEFITEC, Department of Physics, NOVA School of Science and Technology, Universidade NOVA de Lisboa, 2829-516 Caparica, Portugal; t.pivetta@campus.fct.unl.pt; 2 Laboratory of Instrumentation, Biomedical Engineering and Radiation Physics (LIBPhys-UNL), Department of Physics, NOVA School of Science and Technology, Universidade NOVA de Lisboa, 2829-516 Caparica, Portugal; pfr@fct.unl.pt; 3 GNanoBio, School of Pharmaceutical Sciences of Ribeirão Preto, University of São Paulo, Ribeirão Preto 14040-900, Brazil; caroline.botteon@usp.br (C.E.A.B.); pmarcato@fcfrp.usp.br (P.D.M.)

**Keywords:** nanoparticles, phototherapy, cancer, photodynamic therapy, photothermal therapy

## Abstract

Photodynamic therapy (PDT) and photothermal therapy (PTT) are photo-mediated treatments with different mechanisms of action that can be addressed for cancer treatment. Both phototherapies are highly successful and barely or non-invasive types of treatment that have gained attention in the past few years. The death of cancer cells because of the application of these therapies is caused by the formation of reactive oxygen species, that leads to oxidative stress for the case of photodynamic therapy and the generation of heat for the case of photothermal therapies. The advancement of nanotechnology allowed significant benefit to these therapies using nanoparticles, allowing both tuning of the process and an increase of effectiveness. The encapsulation of drugs, development of the most different organic and inorganic nanoparticles as well as the possibility of surfaces’ functionalization are some strategies used to combine phototherapy and nanotechnology, with the aim of an effective treatment with minimal side effects. This article presents an overview on the use of nanostructures in association with phototherapy, in the view of cancer treatment.

## 1. Introduction

Cancer is a leading cause of death worldwide, with an estimated 19.3 million new cases and nearly 10 million deaths caused by cancer in 2020 [1]. During the 20th century, there was an undeniable technological development aiming to enhance the treatment of cancer, mainly regarding to the discovery of chemotherapy. Nowadays, chemotherapy is one of the pillars for cancer treatment, along with surgery and radiotherapy [2,3]. However, it is known that chemotherapy and radiotherapy have severe side effects to the patient, mainly due to the non-specificity of the treatment [4]. Within this context, phototherapy has gained attention as an alternative treatment with reduced side effects [4]. 

Photodynamic therapy (PDT) and photothermal therapy (PTT) are photo-mediated therapies with different damage mechanisms that consist in the generation of reactive oxygen species (ROS) and heat, respectively [4,5]. These effects result in the cells’ death, thereby, with a potential application for treatment of several types of cancer [6]. PDT requires the application of photosensitizer drugs (PS) that will be triggered by radiation.

These drugs generally present poor solubility in physiological conditions, which can impair therapy’s success [7]. For this purpose, it is necessary to find appropriate nanoparticulate systems that can deliver these drugs to the cancer cells. Currently, there is not a unique definition that is accepted internationally, however nanomaterials are often described in the scale of 1–1000 nm [8]. Nanotechnology emerged in order to enhance problems related to drugs’ solubility and provide a targeted treatment, enabling to reduce drugs’ dosage and also minimize several side effects in patients [9]. Additionally, through nanotechnology research, there are several types of nanoparticles, particularly metallic nanoparticles such as gold nanoparticles, that can generate heat upon exposition to light, which can be useful for PTT [10] as it induces hyperthermia in the tumor environment, consequently leading to cancer cells’ death [11]. PTT is a non-invasive and selective technique which can potentially suppress many kinds of tumors [12]. Cancer treatment with the PTT approach offers many advantages, such as sensitization of hypoxic regions, reinforcement of the immune system, releasing of thermo-sensitive substances and increasing susceptibility of cancer cells to chemotherapeutic agents [13]. The combination of PDT and PTT is also possible through the use of a sensitizing agent able to produce ROS and hyperthermia [5]. 

**Figure 1:**
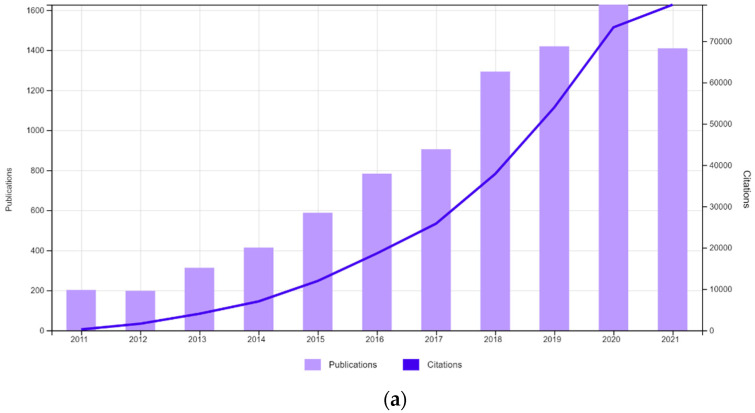

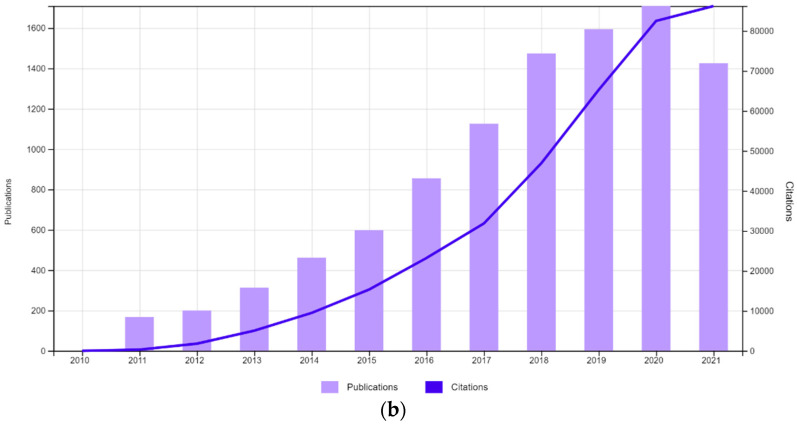
Updated number of publications and number of citations in the last ten years listed in the Web of Science platform using as search topics: (**a**) “Photodynamic Therapy AND Nanoparticles” and (**b**) “Photothermal Therapy AND Nanoparticles” (November 2021).

NPs for phototherapy have been extensively investigated and reported in the literature [14,15], and in this work, new issues concerning NP systems’ design, in view of cancer treatment under photodynamic and photothermal therapies, will be addressed. The referred new issues are intended to exemplify recent approaches related to nanoparticle conditions, such as the targeting of drugs in the tumor site and problems and/or new achievements related to the phototherapy. The overall situation and trend of research in both therapies using nanoparticles is clearly demonstrated in Figure 1, which shows, in the last ten years, both number of publications and number of citations listed in the Web of Science platform using “Photodynamic Therapy AND Nanoparticles” and “Photothermal Therapy AND Nanoparticles” as search topics, where both number of publications and of citations are increasing strongly in recent years.

## 2. Photodynamic Therapy

### 2.1. A Brief Introduction

**Figure 2:**
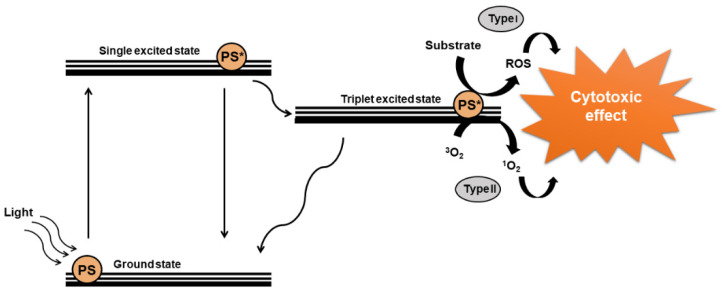
Jablonski diagram representation and the photodynamic therapy mechanism of action.

PDT has been used for centuries, mainly to treat skin disorders, with most treatments involving the intake of extracts of some types of plants followed by exposition to the sun [16]. The main discovery took place in Germany in 1900, where Oscar Raab and Hermann von Tappeiner were investigating the behavior of protozoan *Paramecium* spp. in the presence of the dye acridine orange. They verified that the protozoan died after the exposure to the sunlight coming from an adjacent window. This discovery was important later for the successful achievements on the human skin carcinoma treatment, and by 1904, it was found that the presence of oxygen was important for the treatment, originating the name photodynamic [17]. Currently, PDT is a highly successful and barely or non-invasive type of treatment for several skin disorders, such as psoriasis and cancers [18]. There are three important elements to perform PDT, which are a photosensitizer drug, the light source, and the presence of oxygen. The interaction of these elements results in reactive oxygen species (ROS), which play a key role in the treatment [19]. Upon a specific light wavelength, the photosensitizers (PS) can absorb a photon, which will lead to a conversion from the single basic state to the single excited state, as shown in Figure 2. From there, it can make an intersystem, crossing to a metastable triplet state, which in turn can take two possible paths known as PDT type I or type II. In type I, the activated photosensitizer can trigger a series of reactions with biomolecules generating radicals that interact with oxygen molecules, creating ROS. On the other hand, in PDT type II, the PS by itself can transfer energy directly to oxygen, resulting in ROS molecules [20,21]. Due to their high oxidizing power, ROS molecules have cytotoxic effects, however, due to the short lifetime, the effect of ROS on cell damage will occur around the created species [22]. 

However, PDT has a limited application that can depend on several factors to achieve a successful treatment. A special mention should be given to the light source. This is an important variable to take into consideration because different light wavelengths have different penetration depths in tissues. For example, ultraviolet (UV) light is known to cause several damages in biomolecules, such as the DNA presents low penetration compared to longer light wavelengths [23,24,25,26,27]. Near-infrared (NIR) light, on the other hand, is capable of higher penetration depths, with the capability of generation of local heat even with low energy input. NIR is also safer than UV, which can cause sunburns, inflammation, and even skin cancer [23,28]. 

Another factor that can impair the efficacy of PDT is the hypoxic tumor microenvironment. To overcome this challenge, some strategies involved the elaboration of nanoparticles with molecules such as catalase, that can react and generate oxygen, or hemoglobin and perfluorocarbon, that serve as an oxygen carrier. Therefore, the inclusion of these molecules in nanoparticles is able to improve the PDT efficacy [29]. 

The photosensitizer drugs themselves are another variable that can interfere with the PDT. For example, some PS can present poor solubility under physiologic conditions and impair the correct distribution of the drug to the target tissue, which of course will interfere with the therapy’s success. To circumvent such a drawback, the use of nanoparticulate systems is addressed, enhancing the drug’s solubility and the cellular uptake, and consequently the PDT efficacy [7,30]. Many types of nanoparticles for PDT have been attempted for different types of cancers, and some aspects of nanoparticle systems for PDT will be discussed in this section.

### 2.2. Nanoparticles with Application for PDT

As mentioned before, in phototherapy, the delivery of PS molecules to the target tissue is a relevant issue, as it is in all cases of drug delivery systems. The NP systems to be used should be suitable to release the active components over a defined period of time with control over the nanoparticle size. The raw materials employed, and their biodegradability, is also important to consider for nanoparticles’ preparation. The most common NPs used in PDT are not only organic-based but also inorganic, such as silica and magnetic NPs. In the next sections, some of the best achievements with the use of nanocarriers will be presented.

#### 2.2.1. Organic Nanoparticles

**Figure 3:**
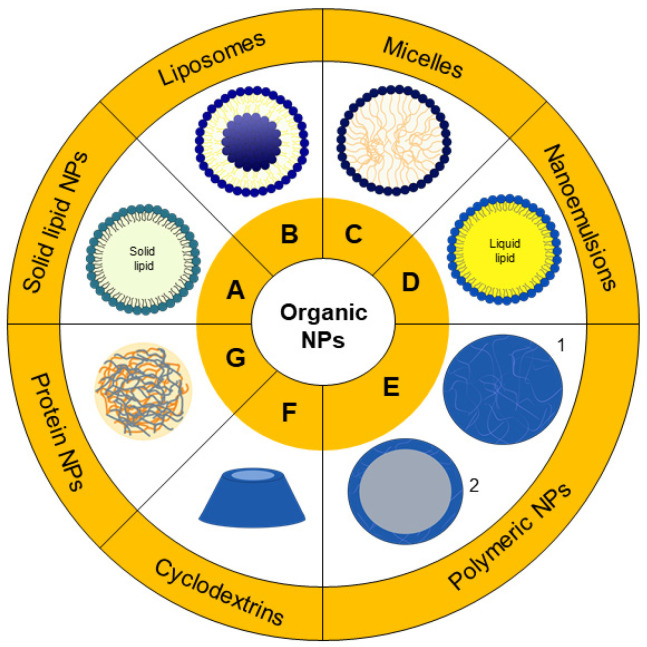
Different organic nanoparticles that can be used for photodynamic therapy: (**A**) solid lipid nanoparticles, (**B**) liposomes, (**C**) micelles, (**D**) nano-emulsions, (**E**) polymeric nanoparticles, (**F**) cyclodextrins and (**G**) protein nanoparticles.

Organic nanoparticles are the most used systems to encapsulate molecules which can be used in PDT. There are several categories based on different materials and respective organization. Figure 3 is a representation that summarizes the most common categories of organic nanoparticles, and in Table 1, there is a brief description with examples of nanoparticulate systems that are cited in this review. 

##### Solid Lipid Nanoparticles

Solid lipid nanoparticles (SLNs) were developed in the 1990s, and ever since, these particles have become the perfect model of safe nanoparticles with an occlusive effect that can also increase the drug permeation in the skin [31]. Generally, these NPs are composed of a surfactant layer, with a lipidic nucleus (Figure 3A, and can be prepared by the Müller and Lucks method based on high-pressure homogenization (1996) or by the microemulsion technique developed by Gasco (1993) [32,33]. 

Either way, SLNs’ production requires lipids that are solid at body temperature, such as some triglycerides or glycerides mixtures. Due to their composition, SLNs are well-tolerated, are biodegradable and can easily be produced on a large scale and at low cost [31,34,35]. However, SLNs present some disadvantages, such as the limited encapsulation efficiency and the possibility of drug release during the storage time. In order to overcome these problems, a second generation of SLNs was developed [34], the so-called nanostructured lipid carriers (NLCs). They consist of SLNs with a less ordered solid matrix based on a mixture of lipids. There are three types of NLCs: imperfect, amorphous and multiple [34]. The imperfect NLCs are composed by a blend of solid lipids with different chain lengths as well as the lipid saturation degree, characteristics that lead to the creation of an imperfect solid matrix. The amorphous type is produced from special solid and liquid lipids, creating a solid particle that does not crystalize. The combination of solid lipids with higher amounts of liquid lipids results in the multiple type, in which there is the creation of oil nano-compartments inside the solid matrix [34,36]. 

Nanostructured lipid carriers with a photosensitizer precursor (5-aminolevulinic acid) were developed by Qidwai and collaborators [37], aiming for use in basal-cell carcinoma treatment. In their study, the nanoparticles exhibited a sustained release profile, higher retention of the drug in the skin layers and enhanced toxicity. Similarly, solid lipid nanoparticles were used to encapsulate curcumin, a natural product with potential in phototherapy application. Curcumin nanoparticles were revealed to enhance drug uptake into the lung cancerous cells and were able to produce ROS under light exposition, thus presenting potential for phototherapy [38].

Most of the studies employing SLNs and NLCs are intended for skin delivery. For example, Goto et al. [39] developed solid lipid nanoparticles containing aluminum chloride phthalocyanine for melanoma treatment. The developed system showed great stability and the measurements of forced stability indicated that the system would be stable for 12 months. In vitro studies showed no toxicity under dark conditions but, when submitted to a light source, the toxicity was seen dependent on the radiation dose. Almeida et al. [40] also encapsulated phthalocyanine in lipid nanoparticle formulations and demonstrated an enhancement of the drug penetration in the skin, when compared to the control group. Interestingly, NLC formulation with higher amounts of the liquid lipid oleic acid showed greater retainment (89.5%) in the deeper skin layers when compared to the NLC with less oleic acid and the solid lipid nanoparticle. In vitro studies carried out on melanoma cells revealed that that the free drug did not lead to cell toxicity under light conditions, probably due to poor accumulation in the cells but, on the other hand, drugs encapsulated in NLC showed a significant reduction in cell viability starting from 0.1 µg/mL. Therefore, the composition of solid lipid nanoparticles is a relevant parameter that can directly result in a higher effect in therapeutics. 

##### Liposomes

Liposomes are formed by auto-organization of phospholipids in bilayers that, in an aqueous medium, tend to fold on themselves, creating vesicles (Figure 3B) [41]. Due to the lipid’s amphiphilic nature, hydrophilic and hydrophobic drugs can be stored in different compartments of liposomes [42]. These vesicles are usually employed as a model in the study of cell membranes, considering the similarity between them, however, liposomes can also be applied to drug delivery [43,44]. The lipid composition provides great biocompatibility, biodegradability and additionally, does not present toxicity [42,45]. The functionalization of these particles with polyethylene glycol (PEG) can lead to the creation of stealth liposomes, that are able to evade from the immune system and increase their blood circulation [46,47]. Other types of ligands can be used in the functionalization, such as antibodies, which in turn manage a robust targeted drug delivery [48]. Due to the system’s versatility, liposomes are great candidates for photodynamic therapy application.

Foscan^®^ is a commercial photosensitizer formulation already approved in Europe for neck and head cancers’ application. The active drug of Foscan^®^, known as temoporfin, also originated Foslip^®^ and Fospeg^®^, which are liposomes formulations [49]. The temoporfin encapsulation in the lipid carriers presents a similar phototoxicity as Foscan^®^ with significantly lower toxicity. Fospeg^®^, a derivative from Foslip^®^ and distinguished by a PEGylation, is able to provide enhanced pharmacokinetics with longer circulation in the blood [50,51]. Studies in HeLa spheroids showed that the drug delivery via liposomes is a way to decrease the drug’s toxicity in the absence of light, increase the cellular internalization and, consequently, PDT effectiveness [49]. Foslip^®^ and Fospeg^®^ are just examples of formulations developed that are currently approved, however many other liposomal systems containing photosensitizers can be explored targeted to different tumor types.

To overcome issues related with low encapsulation efficiency, drug expulsion and quenching caused by molecules’ aggregation, Cai et al. [52] incorporated fluorogens with singular aggregation-induced emission characteristics (AIEgens) in the lipid, creating a conjugate. Liposomes produced from these conjugates (AIEsomes) were able to show a superior ROS production compared to conventional liposomal systems containing photosensitizer molecules. In vitro studies carried out under dark conditions proved that both AIEsomes and conventional liposomes were toxic for a breast cancer cell line, however when irradiated with white light, AIEsomes exhibited more toxicity compared to conventional liposomes. Afterwards, in vivo studies revealed AIEsomes’ ability to target and image in the tumor site, factors intrinsically related to their accumulation mainly in tumors. Furthermore, irradiation of animals after injection of AIEsomes was able to suppress tumor growth and induce necrosis in the tissue, which did not happen to other experimental groups, revealing the potential of liposomes prepared with AIEgen-lipid conjugates for targeted PDT. 

A similar technique was employed by Kim et al. [53] with liposomes prepared from lipid conjugated with pheophorbide A, which were used as photosensitizers aiming for photo-induced immunotherapy in cholangiocarcinoma. Regardless of whether the technique used to exploit photosensitizer incorporation in liposomes consists in a PS-lipid conjugation or encapsulation, these systems have been studied for PDT in several types of cancer, such as gastric, breast, ovarian, liver, skin and others [45,54,55,56,57]. Liposomes’ features provide an extensive range of new possibilities to create therapeutic carriers that can improve PDT.

##### Micelles

Similar to the previous description of liposomes formation, micelles (Figure 3C) are also formed by the self-organization of amphiphilic molecules, and the resultant particle is different from the vesicles because of the different packing parameters [58,59]. The concentration of amphiphilic molecules must reach values above the designated critical micellar concentration (CMC) to form stable micelles, with a confined hydrophobic interior isolated from the aqueous medium. Polymers can also be materials used for micelles’ preparation if the polymers present hydrophobic and hydrophilic segments. Therefore, the choice of the amphiphilic molecule that will be used is important due to different CMC values [60].

Aiming at a dual action of chemo- and photo-therapy in melanoma, Zhang et al. [61] investigated the preparation of micelles from block copolymer for the co-delivery of the classical anticancer agents Doxorubicin and pheophorbide A. These compounds were incorporated in the polymer chain, and the prepared micelles were successfully internalized into melanoma cells with ROS formation induced by light observed in vitro and in vivo. Micelles showed high inhibition of tumor growth, almost twice that of micelles without irradiation treatment, and significantly higher than treatment with only Doxorubicin.

To obtain a target system for ovarian cancer and metastatic melanoma cells, Lamch et al. [62] developed micelles with a di-block copolymer mPEG45-PLLA70 conjugated with folic acid for the encapsulation of the photosensitizer zinc (II) phthalocyanine. Wang et al. [63], in turn, used hyaluronic acid functionalization in micelles containing protoporphyrin IX to target cells with overexpression of CD44 receptors. The in vitro application of these micelles in monolayers and spheroids of human lung adenocarcinoma cells suggested that the enhanced cytotoxicity was due to higher internalization, and the effect of the interaction between the ligand hyaluronic acid and the receptor. Therefore, these studies suggest that micelles’ functionalization can be an approach to enhance photodynamic therapy using this kind of nanostructure.

##### Nano-Emulsions

A nano-emulsion is a mixture of oil and surfactant in aqueous phase, which demands energy to form small droplets of 20–200 nm (Figure 3D) [64]. Nano-emulsions can be employed as a strategy to enhance the bioavailability of several lipophilic drugs. For example, studies by Machado et al. [65] on formulations of nano-emulsions containing curcumin, a natural product, as a photosensitizer drug revealed that curcumin-nano-emulsion was highly phototoxic to breast cancer cells and produced high levels of ROS. Mongue-Fuentes et al. [66] also used natural raw materials for the development of nano-emulsions for PDT. In their work, acai oil was used for the nano-emulsion preparation, which, combined with light irradiation, resulted in 85% death of melanoma cells, results which were also confirmed by animal studies in mice, with a decrease of tumor volume.

##### Polymeric NPs

On the nanotechnology timeline, polymer-based nanoparticles were firstly reported in 1976 [67]. Since then, the great interest in these NPs resulted in the development of several methods to produce polymeric nanoparticles or PNPs (representation of 1-nanospheres and 2-nanocapsules in Figure 3E), such as nanoprecipitation and solvent evaporation. The solvent evaporation method is an example of a two-step procedure where an emulsion is created, homogenized or sonicated, and then an evaporation step is required to remove the organic solvent in which the polymer was dissolved. On the other hand, nanoprecipitation is a one-step procedure where the polymer and drug are dissolved in a solvent miscible in water and dripped in an aqueous solution containing stabilizer. In both methods, organic solvents are employed, and although toxic solvents such as chloroform are no longer used, ether and acetone are currently used for the preparation of nanoparticles. In these cases, evaporation and purification methods are required to remove solvent residues from the dispersion [68,69,70]. 

Eltahan and collaborators developed polymeric nanoparticles co-loaded with NVP-BEZ235 and Chlorin-e6 (Ce6), named NVP/Ce6@NPs [71]. Ce6 was the selected photosensitizer and NVP-BEZ235 was used due to its ability to inhibit the PI3K/AKT/mTOR pathway that is related to tumor progression and proliferation and inhibit the repair of DNA damage in tumor cells. This sophisticated system plus irradiation was able to generate ROS by the Singlet Oxygen Sensor Green method, followed by tests in the triple-negative breast cancer cell line, and by flow cytometry, the authors discovered that treatment with NVP/Ce6@NPs and irradiation presented a fluorescence approximately 5 times greater compared to the control and nanoparticles without Ce6. These achievements showed the effect of a biochemical and PDT combination to treat a severe type of cancer.

**Figure 4:**
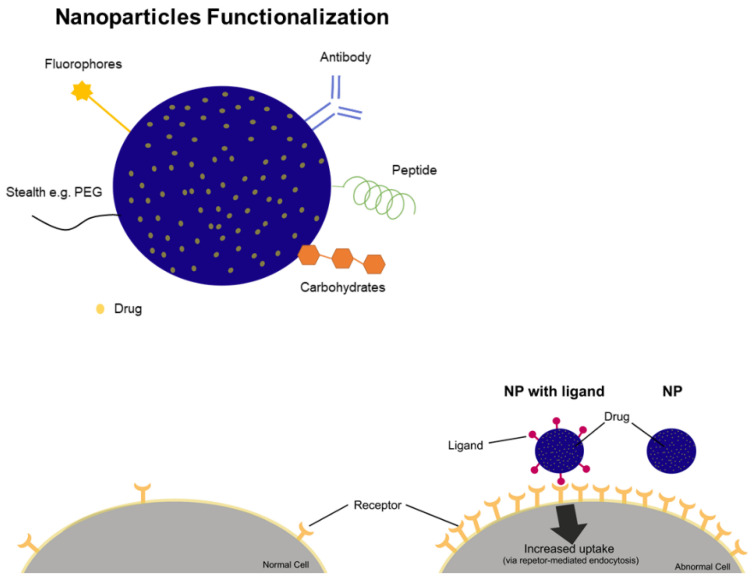
Representation of examples of functionalization to NPs with PEG for stealth NP, with fluorophores for imaging. Functionalization with ligands (e.g., antibody, peptide, carbohydrate and others) can show an advantage in abnormal cells with receptor’s overexpression to enhance uptake by the cells mediated by a receptor endocytosis.

Polymeric nanoparticles can be used to enhance the solubility of drugs as well as to provide drug’s stability and sustained release [72]. PNPs were used to encapsulate the photosensitizer zinc phthalocyanine, and as result, the phototoxicity showed a 500 times increase compared to the free drug in a lung cancer cell line [73]. Polymers’ functionalization is another strategy able to achieve multifunctional nanoparticles [74]. The addition of some type of ligand such as an antibody to the nanoparticle surface allows it to bind specifically to sites where there is an overexpression of the receptor (Figure 4). Transferrin receptors, for example, are overexpressed in breast cancer. Regarding this, Jadia and collaborators [75] functionalized polymers with a peptide (hTf) that is able to bind to transferrin receptor and prepared nanoparticles containing the drug benzoporphyrin monoacid. As expected, functionalized nanoparticles exhibited specificity to the cell line in this study and enhanced the phototoxicity compared to non-functionalized nanoparticles. This successful strategy led to the synthesis of polymers containing different ligands, resulting in nanoparticles with different biological activities such as bioimaging and photodynamic therapy [74]. 

Polyethylene glycol (PEG) has gained attention due to its stealth behavior [72]. PEG has shown promising application due to several properties, namely, inertness in biological systems combined with the non-activation of immune components and low adsorption of biomolecules, such as proteins providing a prolonged circulation in the blood [30,76]. The importance of PEG in PDT was investigated by Yang and collaborators [77] using Ce6 as a photosensitizer, a PDT light source based on a 660 nm laser and synthetized polymers with different densities. It was demonstrated that the drug was detected in the circulation for a prolonged time and a higher amount of Ce6 was detected with high-density PEG nanoparticles. On the other hand, the PDT effectiveness was dependent on the cellular internalization, which is maximized when low-density PEG nanoparticles are applied [77]. Therefore, these achievements debate the need of a parameter’s balance in the design of the nanoparticles to achieve an effective therapy. 

**Figure 5:**
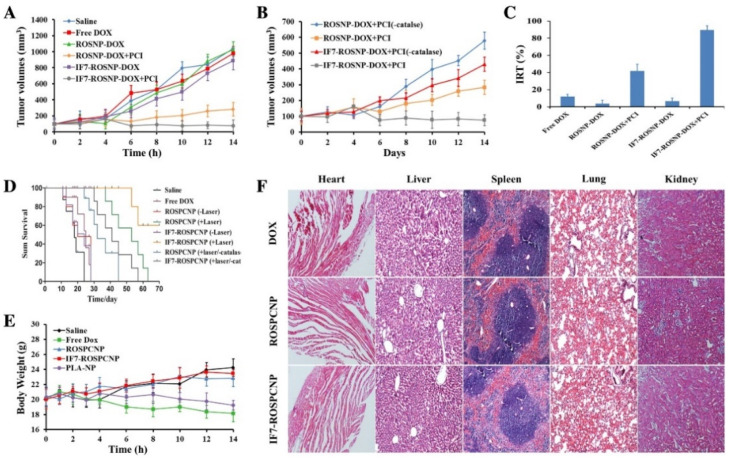
Evaluation of animal studies treated with several samples, such as free DOX, ROSPCNP and IF7-ROSPCNP, in the presence or absence of laser irradiation. (**A**,**B**) Evolution of tumor volume, (**C**) relative inhibition rate of tumor (IRT), (**D**) survival of the animals along the days of the experiment, (**E**) evolution of body weight and (**F**) histopathological analysis of heart, liver, spleen, lung and kidney of animals treated with different approaches (reproduced from Reference [78] with permission from Elsevier. Copyright 2019. Nanomedicine: Nanotechnology, Biology and Medicine).

Studies developed by Luo et al. [78] focused on the development of polymeric nanoparticles with co-encapsulation of Doxorubicin and a photosensitizer. To avoid the known toxicity of Doxorubicin, the strategy used was to link DOX to the polymer, a link that can be cleaved by ROS, and thereby the activation of the nanoparticle is ROS-dependent. They encapsulated the catalase enzyme to act on the intracellular H_2_O_2_ to produce more O_2_ and functionalized particles with a peptide IF7 to target the tumor. This versatile and complex system (IF7-ROSPCNP) was shown to be an effective nanoparticle with accurate tumor targeting, that was able to inhibit tumor growth and prolong survival time when submitted to laser irradiation (Figure 5A–D). Mice treated with ROSPCNP and IF7-ROSPCNP, but not irradiated, were also submitted to histopathological studies, which showed that other tissues were no different from the control group, which suggests that the nanoparticles were safe (Figure 5F).

Deng and collaborators [79] developed systems with tetrakis(4-carboxyphenyl)porphyrin as a photosensitizer, where the drug Doxorubicin was encapsulated forming π-π interactions with PNP to enhance the drug loading. These researchers obtained high drug loading (17.9%) and encapsulation efficiency (89.3%) associated with π-π interactions, as proven by the fluorescence method. Furthermore, in vivo studies showed that the PNPs developed were able to inhibit the growth of breast tumor in Balb/c mice when exposed to laser irradiation. The studies discussed in this topic were a few examples among many reports of photodynamic therapy exploiting PNPs in several types of cancer, such as in cervical adenocarcinoma, glioblastoma, highly aggressive breast cancer and hepatocellular carcinoma, showing the versatility of combining PNPs and PDT for cancer treatment [72,79,80,81].

##### Cyclodextrins

Cyclodextrins (CD) are biodegradable and biocompatible structures composed by oligosaccharides of D(+)-glucose that are able to form nanosized particles by self-organization in aqueous medium [82,83]. As shown in Figure 3F, CD present a conic shape where the hydrophobic cavity provides a way for the solubilization and delivery of hydrophobic drugs [84,85]. The conjugation of the photosensitizer (phthalocyanine) and cyclodextrin was a strategy employed to increase the PS solubility. Assays performed in human bladder cancer cells demonstrated that those conjugates, with higher solubility in water, were more phototoxic to the cells [86]. A similar strategy was adopted by Semeraro and collaborators with a cyclodextrin-chlorophyll *α* conjugate, with a potential photo-induced toxicity in human colorectal adenocarcinoma cells reiterating the versatility of CD-PS complexation for PDT applications [87].

##### Protein Nanoparticles

Proteins are polymeric-type macromolecules formed by repeated amino acid monomers. Due to their biodegradability and low toxicity, proteins gained attention as drug delivery systems, as represented in Figure 3G [88,89]. Recently, Ye and Chi [90] published a review about the recent progress in drug and protein encapsulation. This includes a revision on the different encapsulation techniques, namely, emulsion evaporation, self-emulsifying drug delivery system as well as supercritical fluid, and proposed a novel method using foam that can be quite interesting in the encapsulation. Many types of proteins have been explored for the formation of protein-based nanoparticles, such as albumin. 

Nanoparticle albumin-bound (NAb™) technology was developed to produce albumin nanoparticles. The success of these NPs has already generated a commercial formulation containing paclitaxel, Abraxane^®^, which presented advantages mainly with respect to tumor targeting and drugs’ toxicity decrease [91,92]. In order to be applied to PDT, the association of protein nanoparticles with photosensitizers such as chlorin e6 was investigated by Phuong and collaborators using NAb™ technology [92]. The treatment with the nanoparticles and submission to 660 nm light radiation resulted in a significantly higher toxicity in breast cancer cells and in vivo tumor suppression of 7 times less than the control group, revealing a promising application of protein nanoparticles in PDT.

**Table 1:** Brief description of some organic nanostructures cited in this review as well as their materials, methods of preparation and type of cancer used to test the potential photodynamic therapy of the formulation.

Nanostructures	Materials Employed		Drug	Method of Preparation	Investigated for	Ref.
NLC	Lipid Surfactant	Compritol^®^ ATO 888 Oleic acid Tween^®^ 20	5-aminolevulinic acid	Microemulsion technique	Basal-cell carcinoma	[37]
SLN	Lipid Surfactant	Lecithin Stearic acid Myrj52	Curcumin	Emulsification and low-temperature solidification method	Lung cancer	[38]
SLN	Lipid Surfactant	Compritol 888 CG ATO Stearic acid Sorbitan Isostearate Polyoxyethylene-40 hydrogenated	Aluminum chloride Phthalocyanine	Direct emulsification method	Melanoma	[39]
SLN NLC	Lipid Surfactant	Stearic acid Oleic acid Sodium lauryl sulfate	Chloroaluminum Phthalocyanine	Solvent diffusion technique	Lung cancer Melanoma	[40]
Liposome	Lipid	DSPC DSPG TEL	Curcumin	Thin-film hydration and sonication	Ovarian adenocarcinoma	[45]
Liposome	Lipid Modified Lipid	DPPC Cholesterol DOPE DSPE-PEG-Pheophorbide A	Gemcitabine	Thin-film hydration	Biliary tract cancer	[53]
Liposome	Lipid	DMPC DMPG Cholesterol	Photofrin	Thin-film hydration plus sonication and extrusion	Gastric cancer	[54]
Liposome	Lipid Modified lipid	DPPC Cholesterol DSPE-PEG DOTAP (16:0)LysoPC-BPD	Benzoporphyrin derivative	Thin-film hydration with freeze–thaw cycles and extrusion	Breast cancer	[55]
Liposome	Lipid Edge activator	SPC Sodium deoxycholate	Tetra (4-Tiophenyl) sulfonated phthalocyaninatozinc(II)	Thin film hydration and sonication	Liver cancer	[56]
Liposome	Lipid Surfactant	DOPC DMPC Tween^®^ 20	Zinc phthalocyanine Ruthenium complex [Ru(NH.NHq)(tpy)NO]3+	Ethanol injection method	Skin melanoma	[57]
Micelle	Modified block copolymer	Pluronic^®^ F127-Pheophorbide A	Doxorubicin Pheophorbide A	Thin-film hydration	Melanoma	[61]
Micelle	Modified block copolymer	FA-PEG-PLLA	Zinc(II) Phthalocyanine	Modified dialysis method	Melanoma Ovarian carcinoma	[62]
Micelle	Modified block copolymer	HA-PLGA	Protoporphyrin IX	Solvent dialysis method	Lung cancer	[63]
Nanoemulsion	Lipid Surfactant	Lipoid S100 Poloxamer 188	Curcumin	Interfacial pre-polymer deposition and spontaneous nano-emulsification	Breast cancer	[65]
PNP	Polymer	PEG-b-PLGA	Synthetized zinc phthalocyanine	-	Lung cancer	[73]
PNP	Polymer	PLGA-PEG PLGA-PEG-methoxy PLGA-PEG-maleimide	Benzoporphyrin monoacid	Nanoprecipitation	Breast cancer	[75]
PNP	Polymer	PLGA PEMA PVA	Curcumin	Nanoprecipitation	Glioblastoma	[80]
PNP	Modified polymer	PEGylated Bodipy	Doxorubicin	-	Breast cancer	[81]

DMPC: 1,2-dimyristoyl-sn-glycero-3-phosphocholine; DMPG: 1,2-dimyristoyl-sn-glycero-3-phospho-(1′-rac-glycerol); DOPC: 1,2-dioleoyl-sn-glycero-3-phosphocholine; DOPE: 1,2-dioleoyl-sn-glycero-3-phosphoethanolamine; DOTAP: 1,2-dioleoyl-3-trimethylammonium-propane; DPPC: 1,2-dipalmitoyl-sn-glycero-3-phosphocholine; DSPC: 1,2-distearoyl-sn-glycero-3-phosphocholine; DSPE-PEG(2000): 1,2-distearoyl-sn-glycero-3-phosphoethanolamine-N-[amino(polyethylene glycol)-2000]; DSPG: 1,2-distearoyl-sn-glycero-3-phospho-(1′-rac-glycerol); TEL: tetraether lipids; (16:0)LysoPC: 1-palmitoyl-2-hydroxy-sn-glycero-3-phosphocholine; BDP: Benzoporphyrin derivative; PEG: Poly(ethylene glycol); PLGA: Poly(lactic-co-glycolide); PEG-b-PLGA: poly (ethylene glycol) methyl ether-block-poly (lactide-co-glycolide); PLLA: Poly(L-lactide); FA: Folic acid; HA: Hyaluronic acid; BODIPY: boron dipyrromethene.

#### 2.2.2. Carbon-Based Nanomaterials

Nanotubes, fullerenes and graphenes are among the several carbon-based nanomaterials that became widely explored for medical purposes, mainly due to the π-π interactions in their chemical structure and the ability to produce ROS, as a result of acting as a photosensitizer in PDT [93,94,95]. 

The potential of graphitic carbon nitride nanoparticles in PDT using visible light was analyzed by Heo et al. [95] using cervical cancer cells. Their study showed that the PDT allied with nanoparticles selectively killed more cancer cells than the normal cell lines. Other light sources in the NIR region are also found in the literature to carry out PDT with carbon nanoparticles derived from glucose, which resulted in an efficient ROS production [93]. The surface modification technique can also be employed to bind specifically to receptors that are overexpressed in some cancer cells types, as investigated by Xie and collaborators [96]. In their studies, hollow carbon nanospheres with Doxorubicin presented peptide and hyaluronic acid moieties in the surface to enhance the uptake and damage by dual targeting in a lung cancer cell line.

Carbon dots are carbon-based nanomaterials that can be applied for bioimaging, drug delivery and can also be used for PDT [97]. He et al. [98] designed diketopyrrole-based fluorescent carbon dots and the in vitro and in vivo studies showed that they were able to inhibit the tumor growth when irradiated. 

#### 2.2.3. Silica Nanoparticles

Silica nanoparticles (SNPs) present several advantages that can be useful for the design of nanoparticles for PDT, such as the easy production, possibility of functionalization and to obtain particles with a controlled size [99]. An efficient anti-tumor effect was achieved by Liu et al. [100] when exploring the complex combination of a photosensitizer (rose Bengal), carbon dots and the drug Doxorubicin in mesoporous silica nanoparticles. In their studies, the developed nanoparticle had high drug loading capacity and the problems related to carbon dots and PS aggregation were prevented. This system was also able to produce a higher amount of singlet oxygen compared to PS rose Bengal, and the combination with Doxorubicin provided a synergy between chemotherapy and phototherapy that resulted in a 90% decrease of cell viability. 

The high surface area of silica nanoparticles is another advantage as it allows its modification and functionalization, as demonstrated by the work of Lin and collaborators [101], who developed silica nanoparticles with the PS chlorin e6 encapsulated and a gene plasmid at the surface. Through a photo-induced cleavage of coumarin and detachment of the polycation PDMAEMA, with which the cytocidal gene presented an interaction, the nanoparticles could provide the release of the gene, activation of the PS and therefore a synergistic effect of the gene and phototherapy. 

**Figure 6:**
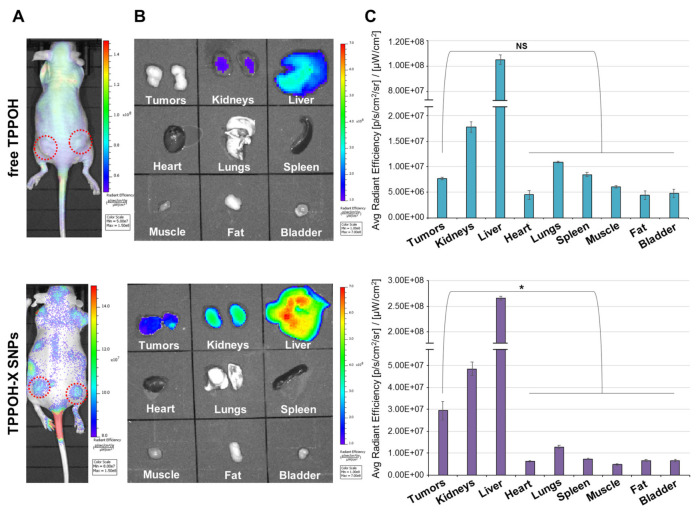
Evaluation of Cy5.5-labeled free TPPOH and TPPOH-X SNPs biodistribution by fluorescence imaging at 24 h post-injection. (**A**) In vivo fluorescence imaging of HT-29 tumor-bearing mice, (**B**) ex vivo fluorescence of tumors and organs, (**C**) fluorescence analysis of tumor and organs. Data are shown as mean ± SEM (*n* = 3). * *p* < 0.05 and NS: not significant (adapted from Reference [102] with permission from MDPI. Copyright 2019, Cancers).

Bretin et al. [102] studied the anticancer potential of the photosensitizer 5-(4-hydroxyphenyl)-10,15,20-triphenylporphyrin (TPPOH) and developed silica nanoparticles coated with the conjugate xylan-TPPOH for photodynamic therapy of cancer. In the xenograft tumor model of colorectal cancer, they studied the biodistribution using Cy5.5-labeled free TPPOH and TPPOH-X SNPs. The fluorescence signal was observed at 24 h post-injection, and as shown in Figure 6A, it was a strong signal for TPPOH-X SNPs, while it showed a minimal accumulation for free TPPOH administration. An ex vivo fluorescence imaging of tumors and organs showed that liver and kidney presented higher intensity compared to the others, but the fluorescence of tumors treated with TPPOH-X SNPs had a superior intensity compared to the other organs when compared to the free TPPOH (Figure 6B). It was also confirmed by a quantitative analysis of fluorescence (Figure 6C).

#### 2.2.4. Magnetic Nanoparticles

Due to their magnetics properties, magnetic nanoparticles can be used in therapy based on the application of an external magnetic field to a targeted tissue. Besides this, it is also possible to attach molecules to it, thus working as a carrier [103]. 

For example, a delivery system prepared with iron oxide magnetic nanoparticles was employed for the targeted delivery of the anticancer Doxorubicin and PDT therapy using a hematoporphyrin. The synergistic effect of PDT with the anticancer drug was shown to provide an effective inhibition of breast cancer in vivo [104]. Recently, Zhang et al. [105] used nanomotors with iron oxide nanoparticles for the delivery of zinc phthalocyanine, and due to the magnetic properties of the iron nanoparticles, the NPs can be targeted to the desired tumor tissue These nanomotors generate O_2_ by catalyzing endogenous H_2_O_2_ for the creation of O_2_ as power to create the nanomotor’s displacement. The system allowed an extended distribution of the photosensitizer as well as ROS generation. Additionally, the generation of O_2_ also supplied an efficient PDT process.

#### 2.2.5. Hybrid Nanoparticles

The hybrid NPs consist in a combination of two or more types of NPs to achieve a unique multifunctional structure [106]. Hybrid NPs composed by the combination of polymers and lipids is a quite common topic found in the literature over the past few years that can also be applied to PDT, as investigated by Pramual and collaborators [107]. In their study, the polymer-lipid-PEG nanoparticles were used for the encapsulation of a PS molecule that exhibited enhanced ROS production and phototoxicity in thyroid cancer cells.

## 3. Photothermal Therapy

### 3.1. A Brief Overview

**Figure 7:**
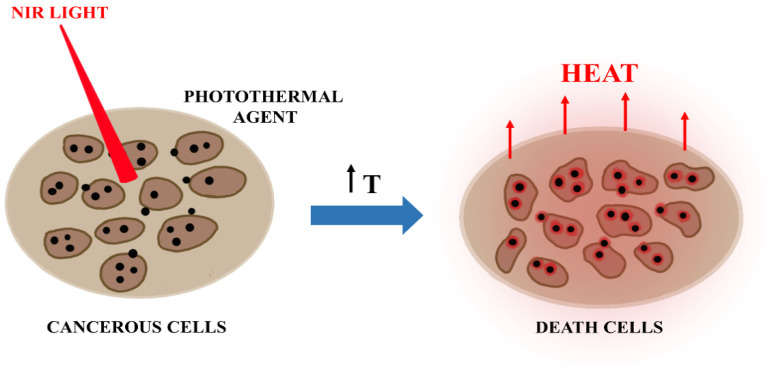
Mechanism of cell death induced by a photothermal agent in the presence of NIR light.

Photothermal therapy (PTT) is a therapeutic strategy using a near-infrared (NIR) laser/light to heat the tumor region and induce cancer cells’ death [108] (Figure 7). Other radiation sources able to generate hyperthermia include visible light, microwaves, radiofrequency and ultrasound waves [109]. PTT has many advantages when compared with conventional therapeutic approaches, including minimal invasiveness and high specificity [110]. In general, PTT approaches explore two mechanisms: The first one involves the exposition of the tumor site to high temperatures (superior to 45 °C) for a few minutes, leading to cellular death by thermal ablation. This approach usually results in stasis in tumor vessels and hemorrhage, which prevent the combination with other treatments. The second one refers to the mild hyperthermia and involves the increasing and setting of temperatures between 42 and 43 °C, prompting cellular damage and enhancing permeability of tumor vessels, which can be used to improve nanoparticles’ uptake by tumors [111,112]. Tumor tissues are more hypoxic and acidic than normal tissues [109]. It is believed that these characteristics make them more susceptible to temperature, thus allowing PTT to selectively destroy cancer cells and protect healthy ones around the tumor [113]. Therefore, since the cancer cells are responsive to this temperature range, this procedure allows the union with synergistic therapies.

PTT displays promising therapeutic efficacy in the treatment of primary tumors or metastasis, in such a way that it has been studied in animal models with various types of cancer, including bone, lung or lymph metastasis [110]. The photothermal effect can also be enhanced using organic dyes or photothermal nano-agents, including metallic nanoparticles, nanocarbons, metal oxide nanomaterials and organic nanostructures [113,114]. 

A synergistic way to improve cancer treatment is its combination with current available therapies, such as chemotherapy, immunotherapy and radiotherapy [109]. The combination of hyperthermia therapy and chemotherapy is commonly explored through hydrophobic interactions, in which nanostructures loading antitumor drugs, such as Doxorubicin and paclitaxel, demonstrated anticancer effects. Moreover, imaging-guided PTT is another improvement to minimize adverse effects and to provide better patient outcomes [115,116], making it possible to plan a therapeutic strategy before and during treatment. 

In the following sections, attention will be given to the recent developments in nanotechnology for photothermal applications of cancer.

### 3.2. Nanoparticles with Application in PTT

#### 3.2.1. Metallic Nanoparticles

##### Gold Nanoparticles

**Figure 8:**
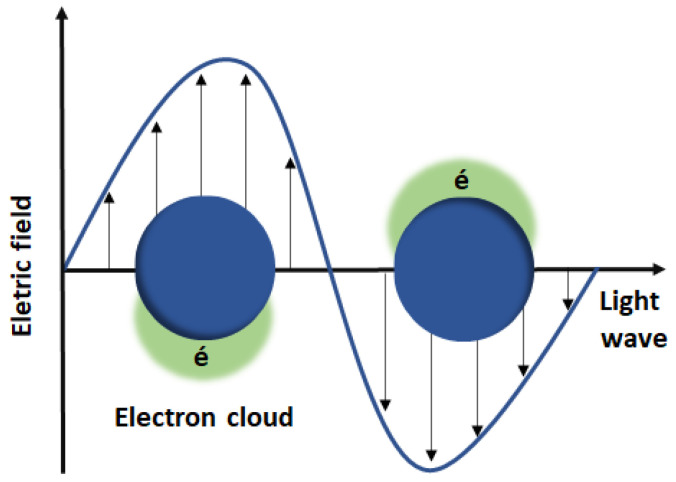
Surface Plasmon Resonance of gold nanoparticles.

Gold nanoparticles (AuNPs) have attracted great interest as photothermal agents for cancer therapy, as they demonstrate efficient local heating after light irradiation [115]. The photothermal conversion phenomenon in AuNPs is based on the collective oscillations of free electrons at AuNPs surface (Surface Plasmon Resonance, SPR) in the presence of electromagnetic radiation (Figure 8). Due to electron excitation and relaxation, this single physicochemical property supplies high localized heating around AuNPs, resulting in destruction of cancer tissues [113,116]. 

It is known that the SPR band of noble metal nanoparticles is much stronger than other metals [117]. Changing AuNPs sizes and shapes, the range of the SPR wavelength of AuNPs is shifted from the visible to the near-infrared (NIR) region, and optical properties can be readily tuned. One of the most interesting parts of the diminished nanoparticles’ diameter is due to the fact that decreasing the size (<5 nm) allows them to be excreted by urine, improving their clearance from the body [118].

**Figure 9:**
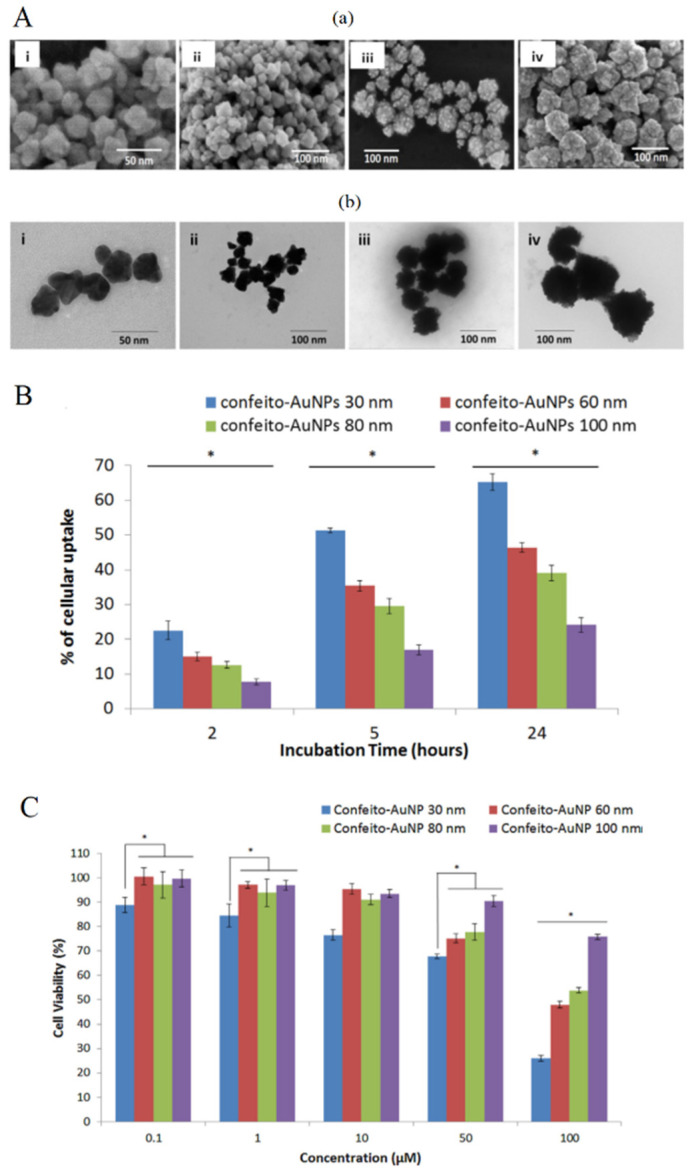
(**A**) Characterization of confeito-AuNPs at (i) 30 nm, (ii) 60 nm, (iii) 80 nm and (iv) 100 nm, by (a) FESEM images and (b) TEM images. (**B**) Evaluation of the cellular uptake of confeito-AuNPs into MDA-MB-231 cells. * *p* < 0.05 (ANOVA). (**C**) In vitro photothermal treatment: MDA-MB-231 cell viability after laser treatment (2 W/cm^2^, 1 min of irradiation) with confeito-AuNPs. * *p* < 0.05 (ANOVA) (adapted from Reference [120] with permission from Elsevier. Copyright 2018, Colloids and Surfaces B: Bio-interfaces).

Moreover, AuNPs size affects the cellular uptake and influences the photothermal conversion efficiency. According to Mie’s theory, smaller nanoparticles show superior heat conversion compared to the larger ones. It was reported that 20 nm gold nanospheres exhibited 97–103% of conversion efficiency [119]. Saw et al. [120] studied the use of four sizes of cystine/citric acid-coated confeito-like gold nanoparticles (confeito-AuNPs) (30, 60, 80 and 100 nm) (Figure 9A) in the photothermal treatment of breast cancer cells. The authors observed that the smallest sizes (30 and 60 nm) of confeito-AuNPs showed higher cellular uptake into MDA-MB-231 cells, compared to larger sizes of AuNPs (Figure 9B). This same size range has been reported in the literature [121]. In vitro studies showed that smaller sizes reached the better PTT cytotoxicity activity against cancer cells (Figure 9C). This result is due to the high surface area in relation to the total mass of NPs, which is observed in smaller nanoparticles. 

Sun et al. [115] employed gold nanoparticle-coated Pluronic-b-poly(L-lysine) nanoparticles (Pluronic-PLL@AuNPs) for the delivery of paclitaxel (PTX) in PTT of solid tumors. The nanoparticles showed efficient photothermal heating capabilities after exposure to an 808 nm NIR laser irradiation and a synergistic effect of chemo-photothermal treatment. The temperature of the PTX-loaded Pluronic-PLL@Au NP-injected tumors increased to 34 °C, which was adequate to eliminate tumors in vivo.

Gold nanoparticles are readily synthesized and allow easy surface modification. Binding a specific ligand on AuNPs surface promotes their targeting to the disease areas and their interactions with cells, such as cancer cells. This procedure increases the effectiveness of the treatment and decreases possible toxic effects in healthy areas of the body [122]. 

One of the strategies of passive targeting to the tumor site is based on the enhanced permeation and retention mechanism that takes place when gold nanoparticles are intravenously administered. However, the AuNPs presence in the blood can arouse attention of the mononuclear phagocytic system (MPS), leading to the rapid elimination of the nanoparticles from the body [123].

The functionalization with polyethylene glycol (PEG) is one of the most effective strategies to optimize the hydrophilic surface and to improve the blood circulation time of nanoparticles [124]. Wang et al. [125] developed PEGylated hollow gold nanoparticles (mPEG@HGNPs) for combined X-ray radiotherapy and photothermal therapy in cancer cells. The in vitro results using the combination of the 808 nm NIR laser and X-ray radiation demonstrated a synergistic antitumor effect with cell viability decreased to 61% and 65% for HGNPs and mPEG@HGNPs, respectively. The nanoparticle cytotoxicity was decreased after PEGylation, due to less mPEG@HGNPs internalized into the cells. Despite that, the targeting enhanced to the tumor site by the mPEG@HGNPs was confirmed using CT imaging in xenografted breast tumor models, due to the EPR effect. 

Cheng et al. [126] reported photolabile AuNPs covalently cross-linkable with a diazirine (DA) terminal group of PEG ligand on the AuNPs surface. The 20.5 nm diazirine-decorated AuNPs (dAuNPs) were obtained after laser excitation at 405 nm. The photothermal therapy in tumor-bearing BALB/c mice was investigated by monitoring the average tumor size in different mice groups. The mice groups that were treated with dAuNPs + λ405 nm and dAuNPs + NIR showed weak tumor inhibition, while the group treated with dAuNPs exhibited a high tumor volume decrease upon 808 nm irradiation (0.75 W cm^−2^). The tumor region reached 26.7 °C after 10 min of light exposure. The PTT efficacy was further confirmed through tissue analysis, which showed extensive necrosis in dAuNPs + λ405 nm + NIR group. 

The extracellular environment of solid tumors has an acidic pH. pH-sensitive AuNPs with potential application in PTT have been reported in the literature [127]. Natural peptides can be used as tumor-targeting agents. Barram et al. [122] used glutathione (GSH), soluble in water, as a coating for AuNPs. GSH is a pH-sensitive polymer, with its isoelectric point (IEP) close to the pH of the cancer cells (~6). Consequently, GSH-AuNPs become responsive to the tumor’s acidic environment, improving its targeting to the desired location. In vitro photothermal therapy was applied to rhabdomyosarcoma (RD) cancer cells, using two types of low-power laser (visible green light (532 nm) and infrared light (NIR) (800 nm)). The study observed cell death values of tumor cells for both types of lasers, and these values were proportional to the longer periods of radiation exposure and, even more so, to the highest concentrations of GSH-AuNPs.

From the molecular point of view, several studies on the effect of nanoparticles on DNA molecules and DNA bases have been performed. These studies clearly demonstrate the damage effect of the gold nanoparticles. Recently, Marques et al. [128] analyzed the decomposition of halogenated nucleobases by Surface Plasmon Resonance excitation of gold nanoparticles. In fact, the halogenated uracil derivatives can be of great interest for cancer therapy [129,130] and the authors demonstrated that the presence of irradiated gold nanoparticles decomposes the ring structure of uracil and its halogenated derivatives with similar efficiency. This decomposition is associated with the fragmentation of the pyrimidine ring, for 5-bromouracil, with cleavage of the carbon-halogen bond, whereas for 5-uorouracil, this reaction channel was inhibited. Locally released halogen atoms can react with molecular groups within DNA, and hence this result indicates a specific mechanism by which doping with 5-bromouracil can enhance DNA damage in the proximity of laser-irradiated gold nanoparticles.

##### Gold Nanorods

Gold nanorods (AuNRs) are one of the many tools employed in cancer photothermal therapy, due to their high capability to transform near-infrared light into heat. The investigation of their aspect ratio allows to adjust a particular SPR band in the NIR [131], consequently reducing damage in normal tissues as these ones have minimal NIR energy absorption. Despite their ability as PTT agents, AuNRs are considered to be toxic to cells, because of the stabilizers, e.g., hexadecyl-trimethylammonium bromide (CTAB), used in their synthesis [132]. Several approaches have been used to minimize the toxicity of AuNRs, such as the binding of polymers to increase their biocompatibility. Kirui et al. [133] improved biocompatibility of AuNRs using poly(acrylic acid) (PAA) for coating of nanoparticles. Liu et al. [134] reduced the toxicity of PEG-AuNRs using multidentate PEG (AuNT-PTP Gm950). 

PTT induced by NIR is known to improve chemotherapeutic efficacy by triggering drug release or increasing the cancer cells’ sensitivity to chemotherapeutics [135]. Hauck et al. [136] revealed that the heat produced by gold nanorods together with the chemotherapeutic drug cisplatin killed 78% more cancer cells than cisplatin alone. Combination therapy can reduce toxicity associated with chemotherapeutics through reducing the effective drug dosage. Duan et al. [137] developed gold nanorods coated with chitosan (CS) derivatives as a carrier of Doxorubicin (DOX) to combine chemical and photothermal effects. In vitro studies demonstrated that these nanoparticles showed low cytotoxicity and potential against cancer cells. Wang et al. [138] developed gold nanorods coated with polydopamine (PDA) and loaded with thiolated poly(ethylene glycol) tumor-homing peptides (NGR and TAT), as a carrier of Doxorubicin. NGR/TAT-DOX-PDA@GNRs allowed a pH-triggered controlled release of DOX and a synergistic effect with the combination of chemo-photothermal therapy. 

Moreover, the efficacy of a targeted synergistic photothermal chemotherapy of breast cancer using gold nanorods (GNRs) functionalized with hyaluronic acid (HA) and folate (FA) to deliver DOX was demonstrated by Xu et al. [139]. The therapeutic system showed a long blood circulation time and high tumor site accumulation. In vitro photothermal chemotherapy was evaluated. Cell viability of MCF-7 cells treated with GNRs-HA-FA-DOX + NIR was reduced to 31%. The authors also investigated the synergistic effect of PTT chemotherapy in vivo and GNRs-HA-FA-DOX exhibited an excellent antitumor effect against MCF-7 breast tumors in nude mice. After 5 min of light exposure, the temperature of MCF-7 breast tumors in nude mice treated with GNRs-HA-FA-DOX reached 67.5 °C (1.5 W/cm^2^), leading to irreversible tumor cell death.

In metastasis, cancer cells migrate and invade the surrounding tissues, and therefore collective cell migration is directly related to cancer aggressiveness. This process involves interactions between neighboring cells through the cell junctions and contraction motions of the cytoskeleton filaments [140]. Studying the migration and invasion of cancer cells, Wu et al. [141] developed AuNRs functionalized with PEG and Arg-Gly-Asp (RGD) peptides. These authors found morphological changes of many cytoskeletal and cell junction proteins after PTT treatment, suggesting that interactions between integrin-targeted AuNRs and cells could trigger inhibition of cancer collective migration. 

It should also be reported that an effective combined therapy of paclitaxel-loaded gold nanorods against head, neck and lung cancer cells was developed by Ren et al. [142]. Paclitaxel was loaded into a hydrophobic pocket of the polymeric monolayer on the surface of NIR-absorbing AuNRs, which allowed the efficiently direct cellular release of the hydrophobic drug via a cell membrane mimicking two-phase solution. It was demonstrated that the PTT approach with this developed nanocomplex led to total eradication of tumor cells at a dosage of 0.5 nm of nanomaterials with low-intensity (0.55 W/cm^2^) NIR light. 

Stimuli-responsive materials have attracted attention due to their capability to control the timed release of the entrapped drugs. Near-infrared light (NIR)-responsive polymers have been used for triggered drug delivery in specific tissues [143]. Hribar et al. [144] reported a NIR light-sensitive polymer−nanorod composite for controlled drug release, in the range of body temperature. As the glass transition temperature is near to the physiological temperature, it can be used to control and improve the release of a molecule. The researchers applied this heating system to trigger release of the Doxorubicin from the nano-system. After NIR light exposure, Doxorubicin-encapsulated microspheres were able to decrease 90% of the activity of T6-17 cells.

##### Gold Hybrid Nanoparticles

Although the research on hybrid nanoparticles to improve the diagnosis and cancer treatment has attracted attention due to its potential use in medicine, its safe application in therapy still remains limited [145,146]. In recent years, it has been reported that the development of iron-gold nanocomplexes are used for the combined PTT with magnetic resonance imaging (MRI). The Au shell composes the light-responsiveness portion, while the iron core can be used to improve the ratio of water molecules’ transverse relaxation, leading to strong MRI signals. Additionally, the magnetic center allows the nanocomplex to be directed to the tumor site by means of a magnetic field [147,148]. 

Dong et al. [149] developed gold-nano-shelled magnetic hybrid nanoparticles functionalized with anti-human epidermal growth factor receptor 2 (Her2) antibodies (Her2-GPH NPs) for multi-modal imaging and cancer treatment. The nanoparticles were produced by loading gold nano-shells with poly (lactic-co-glycolic acid) (PLGA) attached to perfluorooctyl bromide (PFOB) and superparamagnetic iron oxide nanoparticles, and then binding the antibody. Her2-GPH NPs showed high ability as a contrast agent for both ultrasound (US) and magnetic resonance (MR) imaging. The in vitro cytotoxicity studies demonstrated that Her2-GPH NPs specifically promoted Her2-positive human breast cancer SKBR3 cells’ death after NIR exposure. Abed et al. [148] directed Iron (III) oxide–gold (Fe2O3@Au) core-shell nanoparticles to the tumor site through a magnetic field in Balb/c mice bearing a CT26 colorectal tumor model after intravenous administration of the nanoplatform. The in vivo antitumor studies showed the complete tumor growth eradication after magnetic targeting and subsequent NIR eradication.

The toxicity of Au and magnetic nanocomplexes is still concerning. These nanoparticles can lead, among others, to DNA damage, production of free radicals and modification in cell signaling. Additionally, the toxicity can be caused by nanoparticles’ aggregation in biological fluids. Using biocompatible and water-soluble polymers as a coating makes it possible to improve the colloidal stability and decrease nanoparticles’ aggregation, thus diminishing the cytotoxicity. Abedin et al. [150] improved the colloidal stability of Au–Fe_3_O_4_ NPs in aqueous media using poly-l-lysine (PLL) polymer as a surface coating. Additionally, PLL-Au-Fe_3_O_4_ NPs demonstrated cytocompatibility and NIR light absorption ability.

Mesoporous silica nanoparticles (MSNPs) are highly versatile drug carriers due to their biocompatibility and high surface area, consequences of their well-defined internal mesopore structure, varying from 2 to 10 nm in diameter and with large pore volume. Depending on surface charge and nanoparticle size, the characteristics such as nanoparticle cytotoxicity and cellular uptake can change [151]. Yang et al. [152] designed a system composed of ultra-small gold nanoparticles attached to mesoporous silica nanoparticles (MSN) through Au-S bonds. The in vitro studies showed the fast release of DOX upon NIR light irradiation and synergistic cytotoxic effect against A549 cells. 

Gold nanoparticles lose their ability to convert light into heat under repetitive NIR laser irradiation, including gold nanorods that can change their shape and extinction after NIR exposure. Cheng et al. [153] projected gold/mesoporous silica hybrid nanoparticles (GoMe) for lung cancer detection and treatment. This hybrid system has a good photothermal ability and stability, and maintains its capacity of photothermal conversion after repetitive NIR exposures. In addition, 64Cu-labeled GoMe was used to detect lung tumors in vivo through PET imaging, demonstrating to be a potential theranostic system for cancer therapy.

##### Silver Nanoparticles

Silver nanoparticles (AgNPs) are multifunctional materials which have been used for many applications, such as biosensors, electronic compounds, antimicrobials and medicines [154]. Their general use is due to singular characteristics such as size and shape being controllable, easily modified surface and optical and electrical properties [155]. Additionally, their antibacterial activities are widely known [156].

AgNPs can be produced through various physical and chemical methods [157]. Spherical AgNPs are frequently synthesized using the Turkevich method [158] with citrate as a reducing and stabilizing agent or with NaBH_4_ as a reducing agent [159]. In recent years, many researchers are using biological methods to produce AgNPs. These techniques utilize plants, fungi, algae and other organic sources to synthesize nanoparticles with great stability [160].

Application of AgNPs in the biomedical field is still limited due to the concern of their intrinsic toxicity. Interactions of AgNPs with the human body are not yet well-understood [161]. Modifying its surface with biodegradable molecules and polymers or incorporating these nanoparticles into hybrid systems are some of the ways that many researchers have found to increase the biocompatibility of AgNPs. Kim and coworkers [162] developed bovine serum albumin (BSA)-coated silver NPs (BSA-silver NPs) by a single-step reduction process.

Similar to gold nanoparticles, SPR of silver nanoparticles can be tuned to the infrared region by altering their size and shape [163]. Boca et al. [164] designed chitosan-coated silver nanotriangles (Chit-AgNTs) for hyperthermia of human non-small lung cancer cells (NCI-H460) using a 800 nm laser. Wu et al. [165] engineered a nanoplatform for fluorescent probe and label-free imaging of cell surface glycans composed of DNA/silver nanoclusters (DNA/AgNCs) via hybridization chain reaction (HCR). The nanoparticles showed a great ability to convert light to heat, reaching 53.6 °C after irradiation with the 808 nm laser at 1 W cm^−2^ for 10 min. The confocal results demonstrated the applicability of the DNA/AgNCs for labeling glycans on the surface of tumor cells. Moreover, in vivo experiments showed that DNA/AgNCs were able to ablate and inhibit tumor growth under the laser exposure.

PEGylated bovine serum albumin (BSA)-coated silver core/shell nanoparticles loaded with ICG (“PEG-BSA-AgNP/ICG”) were synthesized by Park et al. [166]. These nanoparticles were tested for anticancer activity in B16F10 cells after light exposure. The cytotoxicity results revealed a cell viability of 6% when temperature reached at 50 °C. PEG-BSA-AgNP/ICG also displayed a long plasma half-life, which led to the higher accumulation in the tumor. At 4 h post-administration of PEG-BSA-AgNP/ICG in a B16F10 nude mice model, the tumor temperature reached 49.6 °C with a laser power of 0.95 W. Furthermore, among the treatment groups, the “PEG-BSA-AgNP/ICG + PTT group” was the only one that exhibited significant inhibition in tumor growth.

#### 3.2.2. Carbon-Based Nanomaterials

##### Carbon Nanotubes

Carbon nanotubes (CNTs) are cylindrical structures constructed from a sheet of graphene [167]. These NPs are allotropic forms of carbon, with diameter in the nanometric dimension and various millimeters in length [168]. CTNs are classified into two types, according to the number of layers in their structure: single-walled carbon nanotubes (SWCNTs), which consist of a single graphene sheet, and multiwalled carbon nanotubes (MWCNTs), consisting of several sheets forming concentric cylinders [169].

CNTs have a wide range of properties that make them unique nanomaterials, such as excellent electrical, thermal and optical conduction, mechanical strength [170] and high surface areas, which can be easily functionalized [171]. Indeed, CNTs are usually modified with molecules that help to enhance their biocompatibility or enable specific functions [172]. Attachments to PEG is one of the major types of CNTs’ functionalization to improve biocompatibility, water solubility and stability [173]. Sobhani et al. [174] successfully attached PEG onto the CNTs’s surface.

CNTs have a wide NIR absorption which is dependent on the size and shape of these nanomaterials [175]. Exposing CNTs to NIR light releases vibrational energy in the form of heat, and could be used for cancer cell ablation [176]. The application of CNTs in PTT for the treatment of various kinds of human cancer xenografts in animal models has been investigated in the literature and has been demonstrated to be effective [177].

Li and collaborators [178] designed an interesting system for curcumin (Cur) delivery composed of functionalized single-walled carbon nanotubes by phosphatidylcholine and polyvinylpyrrolidone (SWCNT-Cur). Results of the cellular uptake study showed that SWCNT-Cur effectively improved the delivery of Cur into cells within 4 h. Compared with native Cur, the formulation developed obtained an uptake amount 6-fold higher. Additionally, biodistribution studies demonstrated that SWCNT-Cur could enhance curcumin blood concentration up to 18-fold. Lastly, this system was evaluated for its ability of photothermal ablation in an in vivo model. Among all the groups tested (saline + laser, Cur + laser, SWCNT + laser, SWCNT-Cur + laser), the SWCNT-Cur and laser (808 nm) groups showed the most significant suppression on tumor weight and volume, indicating the synergistic anticancer effect of Cur and PTT.

Waghray et al. [179] synthesized MWCNTs coated with phospholipid-poly(ethylene glycol) and conjugated with an anti-P-glycoprotein (Pgp) antibody, to enhance Pgp-specific cellular uptake. Pgp is an ATP-binding transporter, expressed on tumor cell membranes, and it is related to cancer drug resistance. The phototoxicity of Pab-MWCNTs was investigated in 3T3 and 3T3-MDR1 cells and in a tumor spheroid model (NCI/ADR-RES cells). The nanostructures demonstrated not only Pgp-specific endocytosis by 3T3-MDR1, but they also exhibited dose-dependent phototoxicity only in 3T3-MDR1 cells. Moreover, NCI/ADR-RES spheroids treated with Pab-MWCNTs showed the highest cell death after NIR laser irradiation when compared with control groups.

Zhang and colleagues [180] engineered MWNTs/gemcitabine/lentinan (MWNTs-Ge-Le) to overcome Gemcitabine’s clinical application problems related to short plasma half-life and low cellular uptake. It was observed that the MWNTs-Ge-Le conjugated with rhodamine were internalized by MCF-7 cells about 3 h after incubation. Additionally, encouraged by the results in vitro, the authors evaluated the synergistic antitumor effect of MWNTs-Ge-Le on tumor-bearing mice. MWNTs-Ge-Le nanoparticles have reached the tumor site through the EPR effect. After 3 min of NIR irradiation, the temperature of the tumor surface increased to approximately 42.6 °C, while the PBS group only reached about 36.6 °C. Moreover, it was observed that the size of the tumor significantly decreased, confirming the high synergetic effect of chemotherapy and PTT.

Zhao et al. [181] developed SWCNTs and MWCNTs coated with peptide lipid (PL) and sucrose laurate (SL) (denoted as SCNT-PS and MCNT-PS, respectively), which were conjugated with siRNA (anti-survivin siRNA) for synergistic PTT and gene therapy (GT). The engineered CNTs exhibited excellent temperature-sensitivity and biocompatibility. The effective cellular internalization was confirmed after they observed nanoparticles’ presence in the cytosol of HeLa cells. The in vitro cytotoxicity after 808 nm laser irradiation was evaluated treating cells with 30 μg/mL of SCNT-PS or MCNT-PS. The results showed that 76.2% ± 4.4% and 75.3% ± 3.5% of the cells were led to death by combined therapy in the SCNT-PS and MCNT-PS, respectively. The PT efficacy of CNTs was also evaluated in vivo. After tumor irradiation (1 W/cm^2^ for 5 min), the local temperature reached 42–45 °C. Furthermore, they investigated whether the SCNT-PS/siRNA and MCNT-PS/siRNA complexes could be able to downregulate survivin. The data obtained were promising, indicating that cells that received treatment with SCNT-PS + P + G and MCNT-PS + P + G, followed by PTT, had about a 60% decrease of survivin expression in comparison with GT alone (SCNT-PS + G and MCNT-PS + G).

Besides PTT generating anticancer immune responses, evidence suggests that it could also induce an effect called the abscopal effect [182]. This effect refers to the immune response generated when the primary tumor site is irradiated, which can lead to the regression of metastatic cancer in distant sites that were not irradiated [183]. Nevertheless, some tumors are capable of creating inhibitory binders, which connect to inhibitory co-receptors (immune checkpoints) expressed on tumor immune cells [184]. This activity induces negative regulatory pathways leading to loss of immunological control, allowing tumor growth progression and decreasing immune response to various therapies [185]. New immunotherapeutic approaches are focusing on blocking immune checkpoints in order to recover the suppressed immune response [186]. Among the immune checkpoints, the cytotoxic T-lymphocyte antigen 4 (CTLA-4) is an inhibitory receptor expressed by regulatory and conventional T cells, which suppresses T cell activation via cell intrinsic and extrinsic pathways. Ipilimumab, an antibody against the inhibitory co-receptor CTLA-4, is one of the main targets of immunotherapy [187,188].

CNT-mediated photothermal therapy in combination with checkpoint inhibitors can be used to maximize the abscopal effects of PTT. Li et al. [189] designed SWNT functionalized with glycated chitosan (GC), an immunoadjuvant, for specific treatment of an aggressive 4T1 murine breast cancer model, upon 1064 nm laser irradiation. Putting together SWNT-GC-laser therapy with anti-CTLA-4, they have achieved synergistic immunomodulatory effects, inducing antitumor immune response and an increase of the survival time of the treated mice group (up to 58 days). 

Recently, McKernan and his group [190] presented a delivery nano-system to treat metastatic breast cancer composed of SWCNTs that integrates PT therapy and checkpoint inhibitor immuno-stimulation with anti-CTLA-4. The SWCNTs were functionalized with the protein annexin A5 (ANXA5), which has great affinity to the anionic phospholipid phosphatidylserine expressed on endothelial cells of the tumor vasculature and on tumor cell membranes. The authors noted that PTT with SWCNT-ANXA5 alone was able to destroy primary EMT6 tumors, reaching a temperature of 54 °C at the site, but failed to eliminate the metastasis. On the other hand, the combination of photothermal therapy with SWCNT-ANXA5 and anti-CTLA-4 improved overall survival, leading to 55% of the treated mice surviving at 100 days post-injection. Moreover, in animals who received this combined therapy, increases in the numbers of helper T cells CD4+ and cytotoxic T cells CD8+ were observed, indicating an increase in the immune response.

##### Hollow Carbon Nanospheres

Hollow carbon nanospheres (HCNs) are mesoporous nanomaterials with high pore volume and surface area [191]. Due the carbon chains, a great amount of a hydrophobic drug can be loaded into their structure, making them a potent drug carrier [192]. Similar to carbon nanotubes, HCNs have a great ability to convert NIR light into heat, which can be used to modulate the drug release at the tumor site [193]. 

Wang et al. [194] produced biocompatible HCNs for loading and release of paclitaxel (PTX) and PT therapy. The nanoparticles have demonstrated excellent photostability and ability to effectively release the loaded PTX. Additionally, in vitro experiments showed great thermal ablation of HCT116 cells using 50 μg/mL of HCNs and a 3 W/cm^2^ laser power density for 180 s.

Xu and his group [195] produced a hollow carbon nanosphere capped with olyethylene glycol-graft-polyethylenimine (HPP) as a photothermal agent. Optical properties were investigated using a 1064 nm laser and power density of 0.6 W/cm^2^. After 7 min of laser exposure of the nanoparticle’s dispersion, an increased temperature in the range of 17 to 44 °C was observed, indicating an excellent heat conversion efficiency. The photothermal therapeutic effect in vitro (4T1 cells) and in vivo (Balb/c mice inoculated subcutaneously with 4T1 cells) was also evaluated. The percentage of cell death for in vitro experiment varied from 40% up to 95%, using the HPP concentrations of 10, 20, 40, 80 and 160 µg/mL. 4T1 tumor-bearing mice treated with only 40 µg/mL were irradiated. After 14 days, tumors were measured, showing a significant decrease of volume.

#### 3.2.3. Metal Oxide Nanoparticles

##### Iron Oxide Nanoparticles

Magnetic nanoparticles are mostly formed using magnetite (Fe_3_O_4_), maghemite (γ-Fe_2_O_3_) or a combination of both [196]. Due to their intrinsic magnetic properties (super-paramagnetism), these nano-systems have emerged as potent contrast agents (CAs) for magnetic resonance imaging (MRI) and biomedical purposes [197].

In the field of cancer therapy, magnetic nanoparticles can be specific delivery drugs by application of alternating magnetic fields to targeting tumor sites and eliminating them using localized moderate heating [198].

Cabana and coworkers [199] compared the application of photothermal (PT) therapy using magnetic multicore nanoflowers versus magnetic hyperthermia (MHT) of magnetic nanospheres. The NPs’ performance in MHT and PT was carried out in water and in cancer cells. They found that nanoflowers are heaters that are more effective for both modalities. In the cellular environment, PT showed excellent results at low doses, while MHT was lost for all NPs. Additionally, magnetite nanoflowers demonstrated the highest cellular uptake and the best antitumor activity after the laser exposure (0.3 W/cm^2^).

Liu and collaborators [200] synthesized the polyethylene glycol-coated ultrasmall superparamagnetic iron oxide nanoparticle-coupled sialyl Lewis X (USPIO-PEG-sLex) with excellent photothermal conversion properties. The nanoparticles were applied for MRI and PTT in human nasopharyngeal carcinoma (NPC) xenografts on a mouse model. After 808 nm laser exposure, the cytotoxicity results showed a reduction of viability of NPC 5-8F cells at reasonable concentrations of USPIO-PEG-sLex nanoparticles. Moreover, the NPs were able to inhibit xenografts’ tumor progression after in vivo post-injection.

Iron oxide NPs exhibit excellent photothermal conversion efficiency, good chemical stability and low toxicity in the biological environment [201]. The in vivo application of iron oxide NPs has been approved by the US Food and Drug Administration (FDA) [202]. However, their use in many clinical approaches is limited due to the low tumor delivery efficiency of the NPs [203]. 

Aiming to enhance the tumor delivery of iron oxide NPs, Wang and his group [203] developed an Ac-Arg-Val-ArgArg-Cys(StBu)-Lys-CBT probe coupled with monodispersed carboxyl-decorated SPIO NPs to form SPIO@1NPs. When SPIO@1NPs entered tumor cells overexpressing furin, a reaction chain developed, resulting in SPIO NPs’ aggregates by cross-linking. The self-aggregation of NPs improved their retention in the tumor site, leading to better T2 imaging results and PTT of cancer cells more effective at low doses.

Surface modification of synthetic nanomaterials with biomimetic cell membranes is a smart strategy to make it harmless and invisible to the immune system [204]. Meng et al. [205] employed vesicles formed from macrophage membranes reconstructed to obtain a biomimetic system for cancer phototherapy. These vesicles coated onto magnetic iron oxide nanoparticles (Fe_3_O_4_ NPs) resulting in Fe_3_O_4_@MM NPs exhibited good biocompatibility and light-to-heat conversion efficiency. Cancer targeting of Fe_3_O_4_@MM NPs was confirmed by cellular uptake in MCF-7 cells. The authors also found that the NPs were able to evade from immune cells, and this activity could be related to the presence of cell membrane components on the nanoparticles’ surface. The Fe_3_O_4_@MM NPs were targeted to the tumor site with application of an external magnetic field, in a breast cancer mouse model. The tumor volume was measured after the laser irradiation, reaching a high tumor regression over time. 

Researchers have been employing phytochemical compounds together with magnetic nanoparticles in order to achieve nanomaterials for phototherapy and drug delivery systems [206]. Kharey et al. [207] obtained 15 nm eugenate (4-allyl-2-methoxyphenolate)-capped iron oxide nanoparticles (E-capped IONPs) through green synthesis using a medicinal plant, Pimenta dioica. These NPs showed good biocompatibility in Human cervical cancer (HeLa) and Human embryonic kidney 293 (HEK 293) cell lines, and excellent efficacy of hyperthermia generation upon laser irradiation at NIR wavelength. 

A delivery nano-system composed of R837-loaded polyphenols coating ICG-loaded magnetic nanoparticles (MIRDs) was constructed for spatio-temporal PTT/immunotherapy synergism in cancer. This system inhibited tumor growth, metastasis and recurrence, which resulted in potent anticancer therapeutic effects with few side effects [208]. 

Silica-coated Fe_3_O_4_ magnetic nanoparticles loaded with curcumin (NC) were synthesized by Ashkbar and colleagues [209] for hyperthermia and singlet oxygen production improvement for in vitro and in vivo experiments. Curcumin (CUR) belongs to the polyphenol class of natural compounds, known for its photosensitizing properties and antitumor activities [210]. The PDT was assessed using diode lasers at 450 nm and PTT was achieved by an 808 nm laser. After injection in a breast tumor mouse model, the results showed that CUR + PDT achieved a tumor volume reduction of about 58%, in comparison with the untreated group, while the NC + PDT + PTT group exhibited more than an 80% reduction compared with other treatment groups. The authors found that the NC + PDT + PTT treatment strategy could interrupt the tumor growth until day ten. This result was related to the synergistic effect achieved by hyperthermia plus ROS generation in the tumor site [209].

##### Manganese Oxide Nanoparticles

In recent years, manganese oxide nanoparticles (MONs) have emerged as contrast and photothermal agents due to their low toxicity and good T1-weighted contrast signals, constituting a promising alternative to the traditional PTT agents [211]. 

Xiang and colleagues [212] developed biocompatible and pH-sensitive MnO-loaded carbonaceous nanospheres (MnO@CNSs) for simultaneous PTT and MRI. The mimetic pH-responsive release of Mn^2+^ in the biological environments (pH 7.4, 6.5 and 5.0) was measured. They observed that MnO@CNSs were stable in neutral solution (pH 7.4), while in acidic pH, the nanoparticles quickly released Mn^2+^ ions (pH 6.5 and 5). These data were confirmed in in vivo experiments, which demonstrated that MRT1 signal values were higher in the acid region of the tumors. The MnO@CNSs PTT effect was investigated under irradiation by an 808 nm laser (2 W/cm^2^) for 10 min. The results showed an elevated efficiency of the MnO@CNSs for in vivo tumor ablation, making this system a potent nanotheranostic tool.

##### Molybdenum Oxide Nanoparticles

Molybdenum oxide nanostructures are reported to display good biocompatibility and biodegradability, making them a safe platform for cancer therapy. MoO3 nanoparticles have excellent absorption in the NIR region, and can also generate singlet oxygen under NIR light exposure, which enables their use for both photodynamic and photothermal therapy [213].

Chen et al. [214] synthesized molybdenum oxide (MoOx) nanosheets using the single-pot hydrothermal method and functionalized them with pluronic F127 (MoOX @ F127) to obtain a biocompatible nano-system with pH-dependent degradable properties for chemotherapy and photothermal therapy. It was observed that MoOx @ F127 showed reasonable stability at pH 5.4 and rapid degradation at pH 7.4, indicating that intact nanoparticles could reach the tumor site through the EPR effect. The ability of MoOx @ F127/DOX to kill tumor cells was investigated in MCF-7 cells after 5 min of 808 nm laser irradiation. Cytotoxicity assessment showed that almost 60% of cells died after treatment. Furthermore, in vivo experiments showed that mice injected with MoOx @ F127/DOX had a tumor temperature greater than 50 °C, suggesting high hyperthermic efficiency of the nanoparticles.

##### Zinc Oxide Nanoparticles

The element zinc has diverse medical applications [215]. Zinc oxide (ZnO) shows high chemical stability, low toxicity, optical, electrical and anticancer properties, becoming a potential alternative for PTT [216]. Production of intracellular reactive oxygen species (ROS) is one of the cytotoxic mechanisms of ZnO NPs [217]. Kim et al. [218] applied hybrid nanoparticles composed of ZnO and berberine (BER) for the chemo-photothermal therapy of lung cancer. The in vitro results revealed an effective antiproliferation activity against A549 (human lung adenocarcinoma) cells without severe toxicity signals observed in rats’ blood tests. 

Liu et al. [219] designed a core-shell nanoplatform based on a zinc oxide (ZnO) core and a polydopamine (PDA) shell to combine chemotherapy with Doxorubicin (DOX), gene DNAzyme (DZ) and photothermal therapy. The nanoparticles showed good photothermal conversion and stability after application of the 808 nm laser for 500 s. Additionally, confocal microscopy demonstrated that ZnO@PDA-DOX/DZ could be internalized by cells and consequently could deliver DZ to stimulate gene-silencing activity. Moreover, tumor-bearing mice treated with ZnO@PDA-DOX/DZ exhibited an effective NP accumulation in the tumor site. The tumor tissue achieved a temperature of up to 47.3 °C, leading to death of the cancer cells and inhibition of the tumor growth. Lastly, the authors measured the levels of survivin in the tumor tissue by Western blotting. The results found low levels of survivin, suggesting the triggering of DZ for in vivo gene silencing.

#### 3.2.4. Transition Metal Dichalcogenide Nanomaterials

Molybdenum disulfide (MoS_2_) nanoparticles display several characteristics that make them excellent photothermal agents for cancer therapy, such as biocompatibility, wide surface plasmon resonance, good light-to-heat conversion efficiency and low cost [220]. In 2014, Liu and collaborators [221] pioneered using PEG-functionalized MoS_2_ nanosheets as drug carriers for therapy of cancer. Two-dimensional MoS_2_-PEG nanosheets have achieved excellent synergistic anti-tumor effects in in vivo studies, after intravenous administration of MoS_2_-PEG/DOX. 

Xie and coworkers [222] synthesized egg yolk phospholipid-modified molybdenum disulfide (MoS_2_) as a PTT agent and drug delivery system for MCF-7 cells’ treatment. The lipid layers on the surface of layered MoS_2_ nanosheets were modified to improve the NPs’ stability and the accumulation of the nanocarrier in mice tumors. Additionally, Doxorubicin (DOX) was conjugated with MoS_2_-lipid nanocomposites for synergistic chemotherapy. Ding et al. [223] produced well-dispersed L-cysteine-modified MoS_2_ (MoS_2_-Cys) nanospheres measuring 422 nm in size. MoS_2_-Cys exhibits biocompatible and good photothermal conversion efficiency (35%) upon 808 nm laser irradiation. The in vitro PTT activity of MoS_2_-Cys nanospheres in S180 mouse ascites tumor cells displayed high cytotoxicity, with the IC_50_ value of 2.985 μg/mL. In vivo experiment data demonstrated a remarkable decrease of the tumor volume of the mice treated with MoS_2_-Cys nanospheres coupled with NIR irradiation.

Qian et al. [224] developed titanium disulfide (TiS_2_) nanosheets functionalized with polyethylene glycol (PEG), obtaining a great PTT agent for in vivo tumor ablation. Balb/c mice bearing 4T1 tumors were treated with TiS_2_-PEG, and after 24 h, exposed to an 808 nm laser at 0.8 W cm^−2^ for 5 min. The researchers found that tumors in the mice were completely ablated. Moreover, TiS_2_-PEG nanosheets were tested as a contrast agent in photoacoustic imaging. Strong photoacoustic signals were observed around the mice tumor after injection of TiS_2_-PEG, indicating the efficient accumulation of these nanoparticles at the targeted site.

Cao et al. [225] produced TiS_2_ nanosheets using a human serum albumin (HSA)-assisted exfoliation method, and later, modification with PEGylated folic acid (FA). TiS_2_-HSA-FA showed photothermal conversion efficiency of about 58.9% after NIR laser irradiation. In vitro and in vivo experiments demonstrated TiS_2_-HSA-FA to have a high biocompatibility and specificity for targeting tumors. In vivo synergistic PTT/radiotherapy (RT) evaluation was assessed in a CT26 tumor xenograft model, under 5 min laser irradiation (808 nm, 0.8 W/cm^2^). Researchers found that the highest tumor growth inhibition effect was achieved by TiS_2_-HSA-FA + NIR+RT, suggesting the combined therapy effect.

#### 3.2.5. Other Nanoparticles

Over the years, many kinds of inorganic and organic materials have been employed to build an effective PTT system. Graphene quantum dots (GQDs) have excellent photothermal conversion efficiency, incomparable morphology and ease of functionalization [226]. Fang et al. [227] fabricated graphene quantum dots (GQDs) as a pH-sensitive delivery system for chemotherapeutic drugs inside cancer cells. After their cellular uptake, the nanocarriers released Doxorubicin (DOX) upon laser irradiation and upon acidification of the intracellular environment. Studies in vitro and in vivo demonstrated the targeting of HA-functionalized carriers to the CD44 receptor overexpressing human cervical carcinoma HeLa cells and inhibition of tumor growth.

Phase change material (PCM) is a type of storage material that stores and releases energy in the form of heat [228]. An example of this kind of substance, fatty acid, has been studied in the thermal response to release drugs [229]. Yuan and coworkers [230] fabricated CuS-DOX-MBA@PCM nanoparticles by a nanoprecipitation method. The system was composed of copper sulfide (CuS) and DOX, encapsulated with stearic acid and lauric acid. Due to drug release in physiological conditions, this nanocarrier was used as a photothermal and imaging-guided agent. In vivo results exhibited improved inhibition of tumor growth related to the synergistic effect of 808 nm laser irradiation and antitumor therapy with DOX. 

The potential anticancer activity of selenium nanoparticles has already been described in the literature [231]. Fang et al. [232] designed a combination of chemo- and PT-therapy based on SeNPs to carry both ICG and Doxorubicin (DOX). Additionally, they conjugated two peptides (RC-12 and PG-6) to SeNPs using chitosan (CS) as the linker. These peptides acted as specific tumor-targeting ligands, which helped to improve the cellular uptake of SeNPs-DOX-ICG-RP. The photothermal effect of NPs was confirmed by the raise of temperature to 78.2 °C after NIR irradiation for 100 s (3 W cm^−2^). In vitro experiments demonstrated that SeNPs-DOX-ICG-RP generated ROS in HepG2 cells and promoted an efficient anticancer activity. Mohammadi et al. [233] engineered nanostructures of selenium-polyethylene glycol-curcumin (Se-PEG-Cur) for PTT and sonodynamic therapy (SDT). The nanoparticles showed great photothermal conversion efficiency (16.7%) and ability to trigger ROS production in C540 (B16/F10) cancer cells. The percentage of viable cells after irradiation of the 808 nm laser decreased to 33.9%, while ultrasound waves could reduce viability to 22.9%.

## 4. Final Remarks

**Figure 10:**
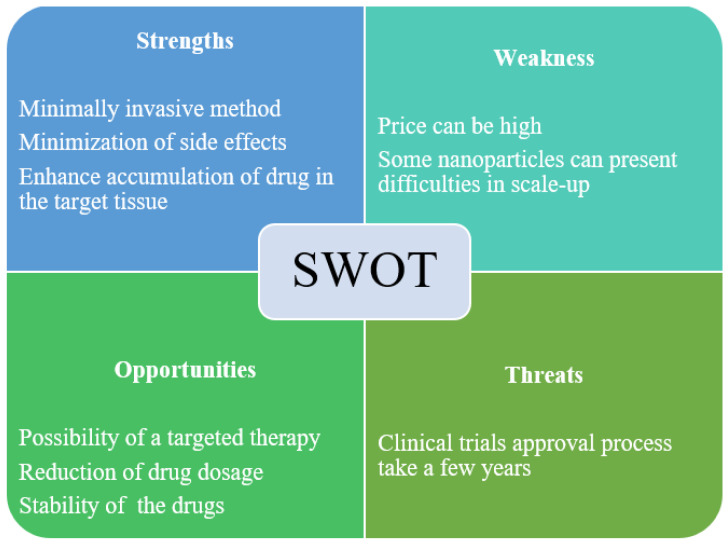
Strengths, weaknesses, opportunities and threats (SWOT) analysis of nanoparticles for Phototherapy.

This review shows that nanoparticles are being extensively investigated for phototherapies nowadays. Regardless of the type of nanoparticle, there a few characteristics, shown in Figure 10, that can summarize the current state of this technology for medical application. The main advantages include the minimally invasive method of therapy, the minimization of side effects and the possibility to target and enhance accumulation of drugs in the tumor. Therefore, it is possible to achieve a targeted therapy with a reduction of drug dosage and greater drug stability. In conclusion, nanoparticle systems are multifaceted structures that are under extensive investigation to create alternatives for conventional therapies of cancer in combination with phototherapy. There are still parameters such as the hypoxic tumor microenvironment that can be an obstacle for PDT, and for phototherapy in general, the limited penetration depth of the light can hinder the use of these systems in cancer therapy. Finally, scale-up and clinical studies are indeed the main challenges in the next few years, however, the incredible diversity of nanoparticles as well as their multiple qualities allied to phototherapy are a promising combination that can result in a more effective and safer treatment for the patients. 

## Data Availability

The data presented in this study are available on request from the cited author.
